# Remimazolam mitigates oxidative stress response in patients undergoing one-lung ventilation

**DOI:** 10.3389/fsurg.2025.1456827

**Published:** 2025-02-21

**Authors:** Shanshan Li, Siriguleng Sana, Dan Wang

**Affiliations:** Anesthesiology Department, First Affiliated Hospital of Harbin Medical University, Harbin, China

**Keywords:** remimazolam, thoracic surgery department, one-Lung ventilation, oxidative stress, inflammatory response

## Abstract

**Introduction:**

The effectiveness of remimazolam in suppressing oxidative stress, inflammatory reactions, and its lung-protective effects in patients undergoing thoracic surgery with one-lung ventilation (OLV) remains unestablished. This study aimed to investigate the protective effects of remimazolam against oxidative stress and inflammatory damage in such patients.

**Methods:**

This randomized controlled trial evaluated 88 patients with lung tumors who underwent single-lung ventilation for thoracoscopic lobectomy under general anesthesia. Patients were randomly divided into an experimental group (R group) and a control group (Con group), with 44 patients in each group. The R group received an intravenous injection of 0.2 mg/kg of remimazolam during anesthesia induction. During anesthesia maintenance, a continuous intravenous infusion of remimazolam at a rate of 0.2 mg/kg/h was administered. The Con group received routine anesthesia. Preoperative and postoperative levels of superoxide dismutase (SOD), glutathione peroxidase (GSH-Px), tumor necrosis factor alpha (TNF-α), and interleukin (IL)-6 were detected. Changes in blood pressure and heart rate were recorded at four time points: upon entering the operating room (T1), immediately after tracheal intubation (T2), immediately after incision (T3), and immediately after surgery completion (T4). The postoperative awakening time, post-anesthesia care unit (PACU) stay time, and frequency of adverse reactions were also recorded.

**Results:**

Postoperatively, the R group exhibited significantly higher levels of SOD and GSH-Px, and significantly lower levels of TNF-α and IL-6 compared to the Con group (*P* < 0.01). At T2, the blood pressure and heart rate in R group were significantly higher than those in Con group (*P* < 0.01), whereas no differences were found between the two groups at other time points. There were no significant differences between the two groups in postoperative awakening time, PACU stay time, or incidence of adverse reactions.

**Discussion:**

Low-dose remimazolam (0.2 mg/kg) can provide protective effects against oxidative stress and inflammatory injuries in patients undergoing OLV, without affecting awakening time or PACU time.

## 1 Introduction

One-lung ventilation (OLV) has become a widely used ventilatory method for clinical anesthesia in cardiothoracic surgery. It is particularly important for the smooth conduct of procedures, such as lung resection under thoracoscopy and radical esophageal cancer surgery. Lung isolation not only prevents contamination of the healthy lung by secretions or purulent blood from the diseased lung, but also prevents collapse of the operated lung, reduces interference with the surgical field, facilitates surgical manipulation, and reduces mechanical injury to the non-resected lung. However, OLV is a non-physiological ventilatory method that can lead to a sudden decrease in the ventilation/perfusion ratio in the non-ventilated lung, resulting in hypoxemia and triggering hypoxic pulmonary vasoconstriction, thereby worsening ischemia in the collapsed lung. During the ischemia of the collapsed lung, the alveolar-capillary barrier can be disrupted, stimulating the release of a large number of pro-inflammatory mediators such as tumor necrosis factor-alpha (TNF-α) and interleukin-6 (IL-6) from alveolar macrophages. This leads to an increase in inflammatory cytokine levels, a decrease in nitric oxide metabolites, an increase in pulmonary capillary permeability, and, ultimately, lung edema, resulting in worsening of lung injury (1–3). Following reventilation of the collapsed lung and reperfusion of the ischemic area, large amounts of reactive oxygen species (ROS) and inflammatory mediators are released, leading to oxidative stress damage and inflammatory responses and resulting in lung ischemia-reperfusion injury (4).

Remimazolam is an ultrashort-acting benzodiazepine sedative. It has the advantages of fast onset, rapid recovery, minimal respiratory and circulatory system suppression, and a low potential for accumulation after long-term use (5). It is currently widely used in painless endoscopy, sedation diagnosis, treatment in intensive care units, and sedation of patients under general anesthesia. Remimazolam primarily acts on gamma-aminobutyric acid (GABA) A receptors. GABA inhibits ROS generation and enhances the antioxidant system, thereby reducing cell apoptosis (6). The GABA signaling pathway also participates in airway inflammatory reactions and inhibits the production of inflammatory factors (7). However, whether remimazolam effectively suppresses oxidative stress and inflammatory reactions has not yet been confirmed. Particularly, there is limited research on the lung-protective effects of remimazolam in patients undergoing thoracic surgery for OLV. Thus, this study aimed to explore the protective effects of remimazolam against oxidative stress and inflammatory injury in patients with OLV undergoing thoracic surgery.

## 2 Materials and methods

### 2.1 Study design and patients

This was a randomized controlled trial, study was approved by the First Affiliated Hospital of Harbin Medical University Ethics Committee (approval number: 202231) and was conducted according to the tenets of the Declaration of Helsinki. Informed consent was obtained from all patients or their family members.

The calculation basis and formula of sample size are $n=\frac{2\sigma^{2}{\left(t_{\alpha }+t_{\beta }\right)}^{2}}{{\left(\mu_{1}-\mu_{2}\right)}^{2}}$. According to the formula *n* = 44. The sample size of the two groups was respectively 44 cases. The total sample size was 88 cases. A total of 88 patients with lung tumors who underwent elective thoracoscopic lobectomy under general anesthesia with OLV between March 2022 and December 2022 at the First Affiliated Hospital of Harbin Medical University were selected. The patients were randomly divided into two groups using the random number table method: an experimental group (R group) and a control group (Con group), with 44 patients per group (*n* = 44). The inclusion criteria were as follows: (1) undergoing elective thoracoscopic surgery with OLV under general anesthesia; (2) age 18–65 years; (3) American Society of Anesthesiologist (ASA) classification grade I–II; (4) cardiac function grade I–II; (5) no significant cardiovascular, pulmonary, hepatic, renal, or other important organ diseases. The exclusion criteria were as follows: (1) severe cardiac, pulmonary, hepatic, or renal dysfunction; (2) immune, endocrine, nervous, or mental system diseases; (3) forced expiratory volume in 1 s <50% in pulmonary function tests; (4) preoperative respiratory tract and pulmonary infections; and (5) history of long-term sedative and analgesic drug use. Data on patient characteristics, including sex, age, height, weight, ASA classification, diagnosis, and medical history, were collected from patient case history.

### 2.2 Surgical protocol

None of the patients were taking any medications preoperatively. After entering the operating room, a radial artery puncture and catheter placement were performed under local anesthesia. Routine monitoring included invasive arterial pressure, electrocardiography, pulse oxygen saturation, end-tidal carbon dioxide pressure, airway resistance, and bispectral index values. In this study, the dosage of remimazolam during the induction and maintenance of general anesthesia was determined in accordance with the instructions. Meanwhile, a pre-experiment was conducted to ensure that patients' blood pressure, heart rate and blood oxygen were stable during the induction and maintenance of general anesthesia, and there was no delay in the awakening time. The study indicates that 0.2 mg/kg of remimazolam used for anesthesia induction and maintenance during general anesthesia is safe and effective, with fewer adverse effects than propofol (8).

During anesthesia induction, the anesthesiologist in the experimental group gave remimazolam at a dose of 0.2 mg/kg intravenous injection, whereas the control group received an equal volume of normal saline using the same method. Both groups were then administered propofol (1–2 mg/kg), rocuronium (1–2 mg/kg), and sufentanil (0.2–0.4 μg/kg). This was followed by double-lumen endotracheal intubation after 4 min using a fiberoptic bronchoscope to adjust the position of the double-lumen tube, which was then connected to the anesthesia machine. The tidal volume was set at 8 ml/kg, and the respiratory rate was set at 12–15 breaths per minute, with an inspiratory-to-expiratory ratio of 1:2. End-tidal carbon dioxide pressure was maintained at 30–40 mmHg. During anesthesia maintenance, the anesthesiologist in the experimental group gave remimazolam at a dose of 0.2 mg/kg/h intravenous injection, whereas the control group received an equivalent volume of normal saline intravenously. Both groups received sevoflurane inhalation continuously, and intermittent intravenous injections of rocuronium (0.2–0.3 mg/kg) and sufentanil were given as needed. The bispectral index values were maintained between 40 and 60 during surgery, and hemodynamic stability was ensured during OLV, with a maintenance of at least 20% above baseline values. Oxygen saturation was maintained above 90%, and airway pressure was maintained below 40 cmH_2_O. After surgery, the endotracheal tube was removed once the patient regained consciousness and recovered spontaneous breathing, and the patient was transferred to the post-anesthesia care unit (PACU).

### 2.3 Observation indicators

The average arterial pressure, heart rate, oxygen saturation, and respiratory rate were recorded at four time points: immediately upon entering the operating room (T1), immediately after tracheal intubation (T2), immediately after skin incision (T3), and immediately after the completion of surgery (T4). The levels of oxidative stress markers, superoxide dismutase (SOD) and glutathione peroxidase (GSH-Px), as well as inflammatory cytokine markers TNF-α and IL-6, were determined using enzyme-linked immunosorbent assay (ELISA) on arterial blood samples (3 ml) collected at T1 and T4. The dosages of sufentanil and propofol, duration of surgery, time to awakening, duration of stay in the PACU, and postoperative complications such as nausea, vomiting, and agitation were recorded.

### 2.4 Statistical analysis

All statistical analyses were performed using SPSS software (version 25.0). Data are presented as mean ± standard deviation. The Shapiro–Wilk normality test was used to assess normality, and Levene's test was applied to evaluate the homogeneity of variance. For comparisons between the two groups, an independent sample *t*-test was used for data that met the assumptions of normality and homogeneity of variance, whereas a corrected Welch *t*-test was employed for data that conformed to normality but not homogeneity of variance. When the data do not conform to normality, the Mann–Whitney *U* test was used. For count data between two groups, the *χ* test was applied. Repetitive data were analyzed using repeated measures of variance. To ensure the accuracy of the comparisons, the Bonferroni correction was applied to adjust for the potential bias resulting from multiple comparisons. The level of statistical significance was set at *P* < 0.05.

## 3 Results

### 3.1 Patient characteristics

The *R* group had a mean age of 53.41 ± 8.61 years, and the average height and weight were 164.89 ± 6.62 cm and 64.64 ± 11.96 kg, respectively. There were 16 men and 28 women. With respect to diagnosis, the R group included 35 patients with lung adenocarcinoma and 9 patients with lung nodules. There were 7 and 37 patients with segmentectomy and pulmonary lobectomy, respectively. The ASA classification was II and III in 28 and 16 patients, respectively. The mean duration of surgery was 234.89 ± 44.53 min. Meanwhile, the Con group had a mean age of 56.09 ± 8.03 years, and the average height and weight were 166.91 ± 7.36 cm and 69.20 ± 10.98 kg, respectively. There were 21 men and 23 women, and 37 and 7 patients had lung adenocarcinoma and lung nodules, respectively. There were 8 and 36 patients with segmentectomy and pulmonary lobectomy, respectively. A total of 27 and 17 patients had American Society of Anesthesiologists (ASA) classifications II and III, respectively. The mean duration of surgery was 217.72 ± 57.39 min. Age, sex, height, weight, clinical diagnosis (lung adenocarcinoma and pulmonary nodules), ASA classification, and surgical duration were not significantly different between the two groups (*P* > 0.05). Further details are listed in Table 1.

**Table 1:** Comparison of patient characteristics between the two groups.

Group	R group (*n* = 44)	Con group (*n* = 44)	Test value	*P* value
Sex (men/women)	16/28	21/23	1.166	0.280
Age (years)	53.41 ± 8.61	56.09 ± 8.03	1.511	0.134
Height (cm)	164.89 ± 6.62	166.91 ± 7.36	1.356	0.179
Weight (kg)	64.64 ± 11.96	69.20 ± 10.98	1.866	0.065
Diagnosis (malignant/benign)	35/9	37/7	0.306	0.580
Surgical method (segmentectomy/pulmonary lobectomy)	7/37	8/36	0.080	0.777
ASA classification (II/III)	28/16	27/17	0.048	0.826
Surgical time (min)	234.89 ± 44.53	217.72 ± 57.39	−1.567	0.121

*P* < 0.01, statistically significant.

R group, experimental group; Con group, control group.

### 3.2 Comparison of oxidative stress response between the two groups

**Figure 1:**
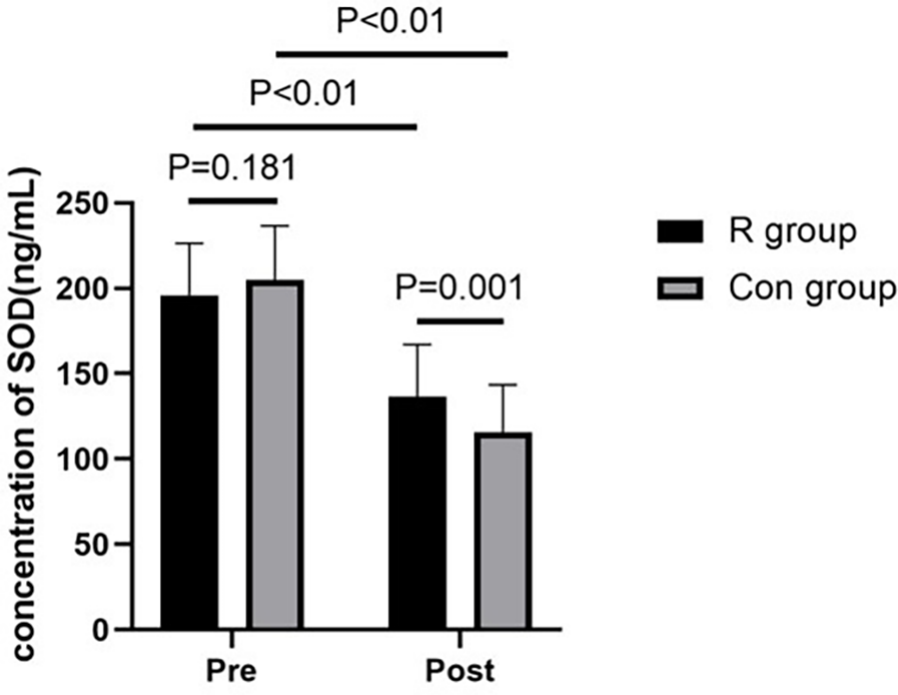
Comparison of SOD levels between the two groups of patients. *P* < 0.01, statistically significant. SOD, superoxide dismutase; R group, experimental group; Con group, control group.

**Figure 2:**
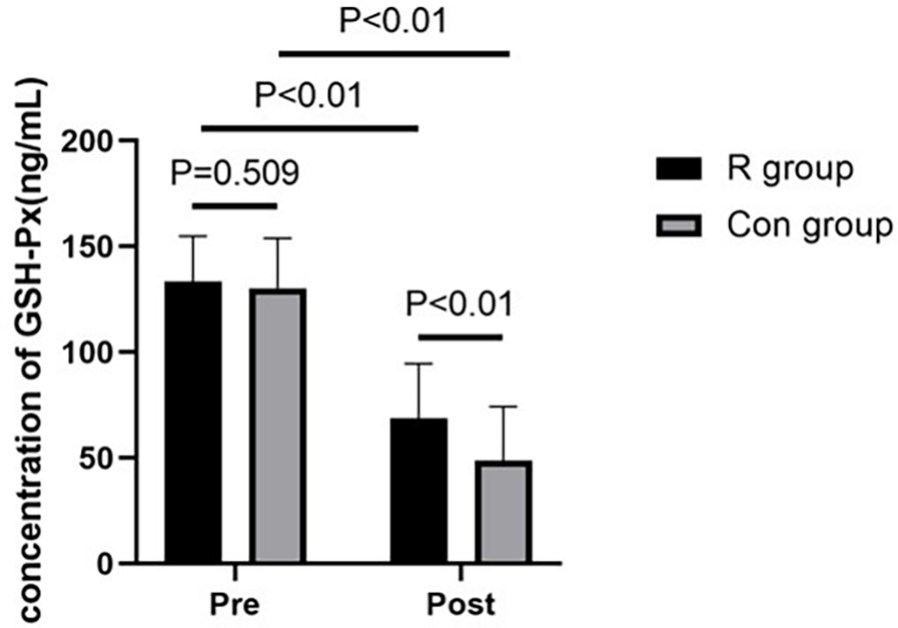
Comparison of GSH-Px levels between the two groups of patients. *P* < 0.01, statistically significant. GSH-Px, glutathione peroxidase; R group, experimental group; Con group, control group.

There were no significant between-group differences in preoperative levels of SOD and GSH-Px (*P* > 0.05). However, postoperative SOD and GSH-Px levels were significantly higher in the R group than those in the Con group (*P* < 0.01). In the R group, the SOD and GSH-Px levels were significantly decreased after surgery than that before surgery (*P* < 0.01). Similarly, the SOD and GSH-Px levels were also significantly decreased after surgery in the Con group (*P* < 0.01). Further details are shown in Tables 2, 3 and in Figures 1, 2.

**Table 2:** Comparison of SOD levels between the two groups.

Indicator	Group	Preoperative	Postoperative	Test value	*P* value
SOD (ng/ml)	R group	195.96 ± 30.46	136.32 ± 30.72	9.592	<0.01
	Con group	204.90 ± 31.70	115.58 ± 27.90	16.556	<0.01
	Test value	1.350	−3.316		
	*P*	0.181	0.001		

*P* < 0.01, statistically significant.

SOD, superoxide dismutase; R group, experimental group; Con group, control group.

**Table 3:** Comparison of GSH-Px levels between the two groups.

Indicator	Group	Preoperative	Postoperative	Test value	*P* value
GSH-Px (ng/ml)	R group	133.38 ± 21.48	68.73 ± 25.85	13.216	<0.01
	Con group	130.18 ± 23.76	48.76 ± 25.44	15.785	<0.01
	Test value	−0.662	−3.653		
	*P*	0.509	<0.01		

*P* < 0.01, statistically significant.

GSH-Px, glutathione peroxidase; R group, experimental group; Con group, control group.

### 3.3 Comparison of serum TNF-α and Il-6 levels between the two groups

**Figure 3:**
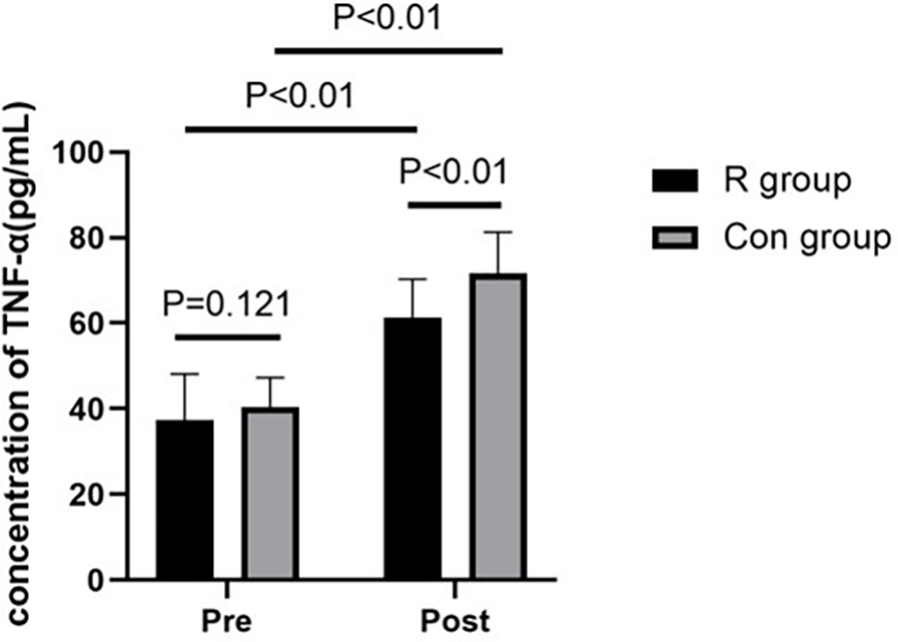
Comparison of TNF-α levels between the two groups of patients. *P* < 0.01 has statistical significance. TNF-α, tumor necrosis factor-alpha; R group, experimental group; Con group, control group.

**Figure 4:**
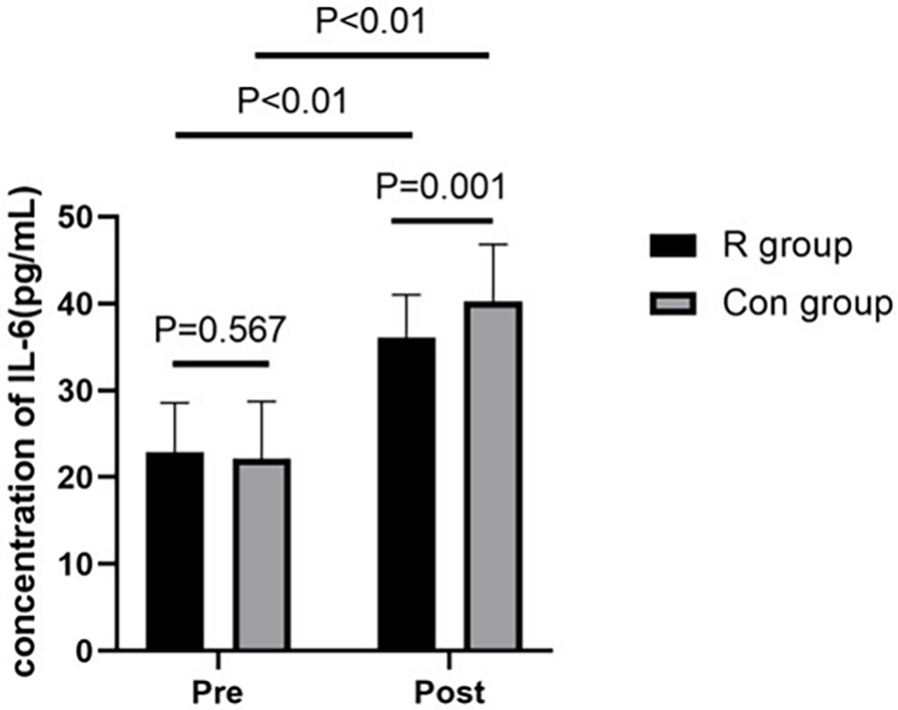
Comparison of IL-6 levels between the two groups of patients. *P* < 0.01, statistically significant. IL-6, interleukin-6; R, experimental group; Con, control group.

The preoperative TNF-α and IL-6 levels were not significantly different between the *R* and Con groups (*P* > 0.05). However, postoperative TNF-α and IL-6 levels were significantly lower in the R group than in the Con group (*P* < 0.01). The TNF-α and IL-6 levels were significantly increased after surgery in both the R group (*P* < 0.01) and the Con group (*P* < 0.01). Further details are shown in Tables 4, 5 and in Figures 3, 4.

**Table 4:** Comparison of TNF-α levels between the two groups of patients.

Indicator	Group	Preoperative	Postoperative	Test value	*P* value
TNF-α (pg/ml)	R group	37.49 ± 10.67	61.22 ± 9.08	−9.807	<0.01
	Con group	40.48 ± 6.77	71.63 ± 9.64	−16.701	<0.01
	Test value	1.569	5.216		
	*P* value	0.121	<0.01		

*P* < 0.01, statistically significant.

TNF-α, tumor necrosis factor-alpha; R group, experimental group; Con group, control group.

**Table 5:** Comparison of IL-6 levels between the two groups.

Indicator	Group	Preoperative	Postoperative	Test value	*P* value
IL-6 (pg/ml)	R group	22.88 ± 5.67	36.10 ± 4.91	−11.717	<0.01
	Con group	22.13 ± 6.61	40.26 ± 6.54	−14.302	<0.01
	Test value	−0.575	3.378		
	*P* value	0.567	0.001		

*P* < 0.01, statistically significant.

IL-6, interleukin-6; R, experimental group; Con, control group.

### 3.4 Comparison of vital signs at different time points between the two groups

Systolic blood pressure, diastolic blood pressure, and heart rate at T1, T3, and T4 were not significantly different between the *R* and Con groups (all *P* > 0.05). At T2, the *R* group had significantly higher systolic blood pressure, diastolic blood pressure, and heart rate than the Con group (*P* < 0.01, Table 6).

**Table 6:** Comparison of vital signs at different time points between the two groups.

Indicator	Group	T_1_	T_2_	T_3_	T_4_
SBP (mmHg)	R group	143.34 ± 20.60	127.80 ± 20.80	117.82 ± 20.17	129.34 ± 22.24
	Con group	143.70 ± 18.66	116.50 ± 17.82	116.75 ± 18.45	129.57 ± 19.40
	Test value	0.087	−2.736	−0.259	0.051
	*P* value	0.931	0.008	0.796	0.959
DBP (mmHg)	R group	79.66 ± 11.47	72.30 ± 11.02	68.30 ± 11.76	76.16 ± 13.91
	Con group	79.52 ± 10.23	65.27 ± 9.52	68.11 ± 10.17	75.25 ± 12.91
	Test value	−0.059	−3.199	−0.078	−0.318
	*P* value	0.953	0.002	0.938	0.751
HR (beats/min)	R group	74.48 ± 13.14	71.30 ± 13.41	63.80 ± 11.83	72.75 ± 12.13
	Con group	75.66 ± 10.92	65.61 ± 11.94	62.11 ± 10.57	73.41 ± 11.74
	Test value	0.459	−2.099	−0.703	0.259
	*P* value	0.648	0.039	0.938	0.796

*P* < 0.01, statistically significant.

SBP, systolic blood pressure; DBP, diastolic blood pressure; HR, heart rate; R group, experimental group; Con group, control group.

### 3.5 Comparison of awakening time and PACU time between the two groups

There were no significant differences in postoperative recovery time and PACU time between the R and Con groups (*P* > 0.05, Table 7).

**Table 7:** Comparison of awakening time and PACU time between the two groups.

Group	Awakening time (min)	PACU time (min)
R group	8.75 ± 4.19	14.00 ± 5.08
Con group	9.57 ± 4.50	13.36 ± 4.94
Test value	0.882	−0.596
*P* value	0.380	0.553

*P* < 0.01, statistically significant.

R group, experimental group; Con group, control group.

### 3.6 Comparison of incidence of adverse reactions between the two groups

There was no significant difference in the incidence of postoperative adverse reactions, including nausea and vomiting, respiratory depression, hypotension, and agitation, between the R and Con groups (*P* > 0.05; Table 8).

**Table 8:** Comparison of the incidence of adverse reactions between the two groups.

Group	NV	RD	Hypotension	Agitation
R group (*n* = 44)	1 (2.27%)	2 (4.55%)	3 (6.82%)	1 (2.27%)
Con group (*n* = 44)	6 (13.64%)	7 (15.91%)	4 (9.09%)	3 (6.82%)
Test value	2.483	1.980	0.156	1.094
*P* value	0.115	0.159	0.694	0.296

*P* < 0.01, statistically significant.

R group, experimental group; Con group, control group; NV, nausea and vomiting; RD, respiratory depression.

## 4 Discussion

This study found that low-dose (0.2 mg/kg) remimazolam to maintain general anesthesia in patients undergoing OLV can increase the levels of SOD and GSH-Px and decrease the levels of TNF-α and IL-6 in patients undergoing lobectomy with OLV in thoracic surgery. These effects may exert a protective benefit against oxidative stress and inflammatory response damage in patients undergoing OLV, providing clinical evidence to inform decision-making in their management. To our best knowledge, this study is the first to investigate the effect of remimazolam on the oxidative stress response in patients undergoing thoracic surgery with OLV and the first study to show that the use of low-dose remimazolam for the maintenance of general anesthesia can increase the levels of SOD and GSH-Px and reduce oxidative stress reactions in these patients.

OLV is a specialized anesthetic technique widely used in thoracic surgery, including lung resection, esophageal cancer, and thoracic aortic aneurysms, due to its better exposure and reduced damage. It relies on mechanical ventilation with a double-lumen endotracheal tube, ventilating one lung while the other collapses. This ensures surgical field exposure and facilitates procedures. However, OLV can cause ventilation/perfusion mismatch, lung injury, ischemia-reperfusion injury, oxidative stress, alveolar and vascular disruption, inflammation, and severe complications (9). Postoperative mortality after thoracic surgery is mainly due to acute lung injury and acute respiratory distress syndrome, with a 40% mortality rate for the latter (10). The pathophysiological mechanism of lung injury during OLV mainly involves ischemia-reperfusion injury (11), particularly in the non-ventilated lung upon re-ventilation and re-expansion at surgery's end due to hypoxia (12). ROS are released early in ischemia-reperfusion (4), while antioxidants like SOD and GSH-Px become depleted, reducing ROS clearance (13). This leads to nuclear and mitochondrial damage, lipid peroxidation, and apoptosis. ROS also induce inflammatory factors like TNF-α and IL-1β (14), activating neutrophils and causing an oxidative stress response in the lungs.

Remimazolam acts on GABA receptors, especially the GABA-A receptor, with high affinity, increasing the frequency of chloride ion channel opening and enhancing the effects of GABA, thereby producing sedative or hypnotic effects (15). GABA can scavenge reactive intermediates produced during lipid peroxidation and readily react with malondialdehyde under physiological conditions, thereby reducing the malondialdehyde reaction, increasing the activity of SOD and GSH-Px, inhibiting the production of ROS, and enhancing the antioxidant system, ultimately reducing cell apoptosis (6). The current study found that SOD and GSH-Px levels were significantly lower postoperatively than that preoperatively in both the R and Con groups, indicating that OLV induced an oxidative stress response in the body. SOD scavenges superoxide anion radicals, whereas GSH-Px promotes the reduction of peroxides. These are important antioxidant enzymes in the body that play a crucial role in the balance between oxidation and antioxidation. In the current study, postoperative serum levels of SOD and GSH-Px were significantly higher in the R group than in the Con group. This indicates that remimazolam attenuates the oxidative stress response in the body and has a protective effect on the lungs. This evidence provides a new direction for the use of remimazolam under general anesthesia.

OLV can lead to a series of pathological and physiological changes that disrupt the alveolar-capillary barrier. It can also stimulate the release of large amounts of pro-inflammatory mediators from alveolar macrophages (1–3). TNF-α and IL-8 are the most important inflammatory mediators in alveolar macrophages and neutrophils. They are mainly involved in immune response and inflammatory response. They are produced by alveolar macrophages; are increased under trauma, inflammation, and other stresses; and can induce the production of other factors such as IL-6. Therefore, the levels of TNF-α, IL-6, and IL-8 can reflect the severity of the inflammatory response caused by surgical stress. Remimazolam is a GABA receptor agonist, and its effect on GABA-A receptors is dose-dependent. At low doses, it has anxiolytic effects, and the sedative and hypnotic effects predominate as the dose increases (6). GABA and its receptors are widely expressed in the airway epithelium and lung tissues and are involved in the pathological processes of respiratory diseases, such as asthma (16), clearance of lung fluid, and airway inflammatory responses (17). The GABA system plays an important role in the regulation of pulmonary fluid balance, and activation of this system significantly increases the rate of lung fluid clearance, improving acute lung injury (18, 19).

The results of the current study showed that compared to preoperative levels, postoperative TNF-α and IL-6 levels were significantly increased in both the R group and Con group. This indicated that OLV surgery can induce the release of a large amount of inflammatory factors in the body. Postoperative TNF-α and IL-6 levels were significantly lower in the R group than in the Con group, demonstrating that remimazolam can reduce the release of inflammatory factors in the body, providing better protection for the lungs, it may be helpful to the prognosis of the operation. However, this study only studied the effect of remimazolam on the inflammatory response after single lung ventilation, and did not explore its long-term effect on the postoperative inflammatory response. Remimazolam improved neurological function and reduced cerebral damage after ischemia/reperfusion, suggesting protection via inhibiting inflammatory factors (20). Septic mice given remimazolam showed higher survival and lower inflammatory mediators compared to controls, indicating its protective effect in sepsis (21). In elderly cancer patients, remimazolam alleviated inflammation and reduced early postoperative cognitive dysfunction (22).

Activation of the GABA receptor by remimazolam leads to the influx of chloride ions into the cell, subsequently reducing excitability and inhibiting neuronal electrical activity, thereby producing a sedative effect (23). The current study found no significant differences in blood pressure and heart rate between the Con group and R group at T1, T3, and T4, except at T2 where the R group had higher values. This suggests remimazolam's mild effect on circulation and safety in OLV patients. This is consistent with the findings of other researchers. In cardiac valve replacement surgery, remimazolam resulted in lower hemodynamic fluctuations, hypotension, and norepinephrine usage compared to propofol (24).

In out study, no significant differences in awakening time and PACU stay were found between the R group and Con group in OLV patients. This phenomenon has also been observed in other studies. For gastroscopy patients, remimazolam's sedative efficacy was comparable to propofol, with faster alert recovery in the remimazolam group. These results confirm remimazolam's safety and effectiveness, without adverse effects on awakening or prognosis (25). Low-dose remimazolam as an auxiliary anesthetic may explain the absence of differences. Postoperative adverse reactions were similar between groups, supporting remimazolam's safety for anesthesia maintenance in OLV patients. Another study found remimazolam non-inferior to propofol for anesthesia induction and maintenance, with significantly fewer adverse reactions in the remimazolam group, indicating good tolerability (26).

The limitations of this study include its small sample size and the single-center design, which limit the representativeness of the research findings. In the future, we will conduct a large sample multi-center randomized controlled trial. The study only used the enzyme-linked immunosorbent assay method to detect the levels of SOD, GSH-Px, TNF-α, and IL-6, and the experimental methods were relatively simple. This study focused only on clinical experiments and did not investigate the mechanism of action of remimazolam. The mechanism of remimazolam can be studied in the future.

In summary, low-dose remimazolam can enhance SOD and GSH-Px levels while reducing TNF-α and IL-6 levels, thereby preventing oxidative stress and inflammatory response damage. Additionally, it does not compromise hemodynamic stability, recovery time, or PACU duration, and exhibits fewer adverse reactions. Therefore, low-dose remimazolam can provide lung-protective effects for patients undergoing OLV during the induction and maintenance of general anesthesia.

## Data Availability

The original contributions presented in the study are included in the article/Supplementary Material, further inquiries can be directed to the corresponding authors.
